# Learning to Swim

**Published:** 1884-09

**Authors:** 


### LEARNING TO SWIM.

THE Process of teaching is a very simple and easy one. When the pupil presents herself and has donned her bathing suit which consists of a sack, skirt, and broad trousers, she is taken to the preparatory room and is taught the proper motions of her arms and legs on a carpet; these mastered, she is taken to the bathing pool, where a strap, so padded as not to hurt her, is passed around her body, and she is placed in the water with her face down, and kept afloat by a rope passed through a pulley. Here she goes through the motions of swimming, which, like her music lessons at home, are indicated by the voice of the female teacher, who counts one, two, three, in a monotone that gives the time to the motions of the limbs.

The next stage is swimming with a float or life-preserver around the body. In this, the action of the limbs is perfectly free, and the pupil, accompanied by the teacher, often succeeds in making a round of the bath in the second or third lesson. All her motions are closely watched, and her attention is sharply called to any false stroke or laggard movement. The motions once perfectly learned, and the pupil soon gathers confidence in her ability to swim, and it is only in a few cases that the float cannot be dispensed with, after the fifth lesson, and the young lady sent out to swim without any other aids than those given her by nature. Girls are taught the same stroke as boys, but there appears to be an essential difference between them in the matter of using the propelling power of the lower limbs. The boy is more vigorous and more propulsive in his legs than in his arms, while with the girls the reverse is the case. Many of the lady swimmers dispense with the skirt, which somewhat retards their motions, and wear simply the sack and trousers. It seems to be the most reasonable swimming costume, for the skirt is apt to hold the water and lessen the speed of the swimmer, by giving her a heavier load to carry.

### THE SPARROW AND THE CAT.

THEY say: A cat once caught a sparrow and was about to devour it when the sparrow spoke up and said, "No gentleman eats till he has first washed his face." The cat was struck with this sage remark, sat the sparrow down and began to wash his face with his paw. The sparrow flew away. This enraged pussy, and he swore —"As long as I live I will eat first, and wash my face afterwards." And cats have done so, ever since.

